# Open communication between patients and relatives about illness & death in advanced cancer—results of the eQuiPe Study

**DOI:** 10.1007/s00520-024-08379-5

**Published:** 2024-03-06

**Authors:** Michelle Haaksman, Laurien Ham, Linda Brom, Arnold Baars, Jean-Paul van Basten, Ben E. E. M. van den Borne, Mathijs P. Hendriks, Wouter K. de Jong, Hanneke W. M. van Laarhoven, Anne S. R. van Lindert, Caroline M. P. W. Mandigers, Annemieke van der Padt-Pruijsten, Tineke J. Smilde, Lia C. van Zuylen, Liesbeth M. van Vliet, Natasja J. H. Raijmakers

**Affiliations:** 1 Department of Health, Medical and Neuropsychology, University of Leiden, Leiden, the Netherlands; 2 Department of Research & Development, Netherlands Comprehensive Cancer Organization (IKNL), PO box 19079, 3501 DB Utrecht, the Netherlands; 3 Netherlands Association for Palliative Care (PZNL), Utrecht, the Netherlands; 4 Department of Internal Medicine, Hospital Gelderse Vallei, Ede, the Netherlands; 5 Department of Urology, Canisius Wilhelmina Hospital, Nijmegen, the Netherlands; 6 Department of Pulmonology, Catharina Hospital, Eindhoven, the Netherlands; 7 Department of Medical Oncology, Northwest Clinics, Alkmaar, The Netherlands; 8 Department of Pulmonology, Hospital Gelderse Vallei, Ede, the Netherlands; 9 Department of Medical Oncology, Amsterdam University Medical Center, Cancer Center Amsterdam, University of Amsterdam, Amsterdam, the Netherlands; 10 Department of Pulmonology, University Medical Center Utrecht, Utrecht, The Netherlands; 11 Department of Medical Oncology, Canisius Wilhelmina Hospital, Nijmegen, The Netherlands; 12 Department of Internal Medicine, Maasstad Hospital, Rotterdam, The Netherlands; 13 Department of Medical Oncology, Jeroen Bosch Hospital, ‘s-Hertogenbosch, The Netherlands

**Keywords:** Bereavement, Cancer, Communication, Oncology, Palliative care, Relatives

## Abstract

**Objective:**

To assess the degree of openness of communication about illness and death between patients with advanced cancer and their relatives during the last three months of the patient’s life, and its association with relatives’ characteristics and bereavement distress.

**Methods:**

We used data from bereaved relatives of patients with advanced cancer from the prospective, longitudinal, multicenter, observational eQuipe study. Univariate and multivariable linear regression analyses were used to assess the association between the degree of openness of communication (measured using the validated Caregivers’ Communication with patients about Illness and Death scale), the a priori defined characteristics of the relatives, and the degree of bereavement distress (measured using the Impact of Event Scale).

**Results:**

A total of 160 bereaved relatives were included in the analysis. The average degree of open communication about illness and death between patients with advanced cancer and their relatives was 3.86 on a scale of 1 to 5 (*SE*=0.08). A higher degree of open communication was associated with a lower degree of bereavement distress (*p*=0.003). No associations were found between the degree of open communication and the relatives’ age (*p*=0.745), gender (*p*=0.196), level of education (*p*>0.773), (religious) worldview (*p*=0.435), type of relationship with the patient (*p*>0.548), or level of emotional functioning before the patient’s death (*p*=0.075).

**Conclusions:**

Open communication about illness and death between patients and relatives seems to be important, as it is associated with a lower degree of bereavement distress. Healthcare professionals can play an important role in encouraging the dialogue. However, it is important to keep in mind that some people not feel comfortable talking about illness and death.

**Supplementary Information:**

The online version contains supplementary material available at 10.1007/s00520-024-08379-5.

## Background

The diagnosis and treatment of advanced cancer have a significant impact on patients and their relatives. Relatives are often involved in the care of patients with cancer, a role that can be fulfilling but can also affect the well-being of the relatives involved. Studies have shown that many relatives experience high levels of caregiver burden, psychological distress and poor quality of life while providing care [1–5]. Even after the death of a loved one, relatives experience psychological problems such as bereavement distress [6]. It is therefore important to explore ways to reduce the burden on relatives, including investigating factors such as open communication about illness and death.

Open communication with a seriously ill person refers to honest and straightforward conversations about illness and death, including the verbalization of associated fears and emotions [7]. Open communication about illness and death between patients and relatives can positively affect the well-being of relatives during caregiving, as it has been shown to be associated with reduced caregiver burden [8, 9] and increased personal relief [10]. Conversely, research has shown that poor communication during the caregiving period is associated with higher levels of depression [11], increased feelings of guilt and regret [12], and more complicated grief experiences [11, 13] among relatives during the bereavement period. These results suggest that communication plays a role in influencing the emotional well-being of relatives following the patient's death [14, 15].

Whether or not open communication between patients and relatives occurs, might be depending on several factors. Firstly, from a broader perspective, religion and culture may influence communication about illness and death. For example, in some religions, life is seen as a sacred gift and it is considered more important to keep the faith and to fight the disease then to talk about incurability of an illness [16, 17]. In addition, in some Eastern countries, death is considered a social taboo, whereas in most Western countries, open communication about illness and death is more accepted [18–20]. However, even within Western countries, there are differences in communication about illness and death. For instance, end-of-life discussions between patients and doctors are more common in the Netherlands compared to Belgium, Spain, and Italy [21]. Secondly, sociodemographic characteristics may play a role. Some studies suggest that female relatives communicate more than male relatives when interacting with patients [7, 22, 23], although other studies found no significant gender differences [24]. A higher degree of open communication seems also related to younger age, higher levels of education, lack of (religious) worldview, and lower levels of depression, but these findings also vary across studies [7, 24, 25].

In summary, although previous studies have examined some aspects of open communication about illness and death between patients and their relatives, it remains difficult to generalize the findings to all settings due to the small number of studies that have focused on the patient-relative communication. To gain more insight into the communication between patients and their relatives in the Netherlands, the aim of this study was threefold: 1) to assess the degree of openness of communication about illness and death between patients with advanced cancer and their relatives during the last three months of the patient’s life, 2) to examine its association with relatives’ characteristics, and 3) to examine its association with relatives’ bereavement distress. The results can be used to create awareness about the current clinical practice and potential benefits of open communication between patients and families and identify subgroups that might need more support to communicate openly with their relatives.

## Methods

### Study design and ethics

This study is part of a larger Dutch prospective, longitudinal, multicenter, observational cohort study on the experienced quality of care and quality of life of patients with advanced cancer and their relatives (eQuiPe study) using an online or paper survey design. Patients and relatives were invited to complete a questionnaire every three months until the patient's death. Three to six months after the patient’s death, the relatives received a final questionnaire. In this study, we used the questionnaires completed within six months of the patient’s death and the final (post-bereavement) questionnaire. The eQuiPe study was exempted from full medical ethical review by the Medical Research Ethics Committee of the Antoni van Leeuwenhoek Hospital (METC17.1491), in accordance with the Dutch Medical Research Involving Human Subjects Act (WMO). Detailed information on the study has been published elsewhere [26]. No funding was received for conducting this study.

### Study population

Patients with advanced cancer were recruited between November 2017 and March 2020, and all patients were asked if a relative (aged ≥ 18 years) was also interested in participating in the current study. Patient-selected relatives were contacted by telephone to provide information about the study. Relatives could include not only partners or children, but anyone closely related to the patient.

### Data collection

Informed consent was obtained from all study participants before completing the paper or online questionnaires via the Patient Reported Outcomes Following Initial treatment and Long-term Evaluation of Survivorship (PROFILES) registry [27]. Patient clinical data were obtained by linking the information to the Netherlands Cancer Registry (NCR). The NCR includes all newly diagnosed malignancies in the Netherlands since 1989. The Netherlands Comprehensive Cancer Organization (IKNL) manages and hosts the NCR.

## Measures

### Openness of communication about illness and death with the patient

Openness of communication about illness and death, as perceived by the relative during the last three months of the patient’s life, was measured using the validated Caregivers’ Communication with Patients about Illness and Death (CCID) scale [28]. The measure consists of five statements, e.g.: “I was afraid to talk with the patient about continuing my life without him/her.” Agreement with each statement was rated on a five-point Likert scale, ranging from 1 (strongly disagree) to 5 (strongly agree). Total scores were calculated as the average of item responses (range 1.0 to 5.0) and inverted so that higher scores represented a higher degree of open communication. These data were gathered in the final post-bereavement questionnaire.

### Bereavement outcomes

Bereavement outcomes were measured using the validated Impact of Event Scale (IES) [29]. The IES assesses the degree of distress in response to trauma or loss and consists of fifteen items, e.g.: “Any reminder brought back feelings about it.” The items were scored on a four-point scale: 0 (not at all), 1 (rarely), 3 (sometimes), and 5 (often). The total score for distress ranged from 0 to 75, which can be divided into 4 categories: subclinical (0 to 8), mild (9 to 25), moderate (26 to 44), and severe (+44) [30]. Higher scores on the IES represented a higher degree of bereavement distress. These data were collected in the final post-bereavement questionnaire.

### Relatives’ characteristics

Relative characteristics included age, gender, level of education, (religious) worldview, relationship with the patient and nationality, all of which were self-reported. Level of education was categorized according to International Standard Classification of Education guidelines: Low: no education, pre-primary, primary, lower secondary education, compulsory education, initial vocational education. Medium: upper secondary general education, basic vocational education, secondary vocational education, post-secondary education. High: specialized vocational education, university/college education, (post)-doctorate and equivalent degrees [31]. Worldview was categorized as having a (religious) worldview (such as Catholic, Protestant or humanist) or not having a (religious) worldview. Relationship with the patient was categorized as partner, child, or other (such as other family members or close friends). Emotional functioning before bereavement was assessed using the emotional functioning subscale of the European Organization for Research and Treatment of Cancer Quality of Life Questionnaire C30 (EORTC QLQ-C30) [32]. This subscale consists of four items [32], and agreement with each item was rated on a four-point scale from 1 (not at all) to 4 (very much). Scores were transformed to a 0–100 scale and dichotomized into low and high emotional functioning using the thresholds established by Giesinger et al. [33]: high (>71) or low (≤71). Higher scores indicated better emotional functioning. The most recent pre-bereavement questionnaire, taken within six months before the patient’s death, was used to assess emotional functioning.

### Statistical analyses

First, we used descriptive statistics to provide an overview of the characteristics of our study population. To address Aim 1, descriptive statistics were used to examine the relatives’ experiences with open communication about illness and death. Second, to address Aim 2, univariate and multivariable linear regression analyses were performed to assess the associations between the a priori selected characteristics of the relatives and the degree of open communication. Categorical variables were included using dummy coding. Finally, to answer Aim 3, a linear regression analysis was performed to assess the association between the degree of open communication and the degree of bereavement distress, adjusting for confounders (gender, age, education, (religious) worldview, relationship with the patient, and level of emotional functioning). Multiple imputation was used to complete the missing data, as Little's test indicated that the data were missing completely at random. All variables had less than 5% missing, except for emotional functioning before the patient’s death (11% missing). Twenty datasets were imputed using multiple imputation by chained equations. The imputation model consisted of the variables included in the analysis model, namely, age, gender, level of education, (religious) worldview, type of relationship with the patient, and level of emotional function before the patient’s death. Estimates of the parameters for each imputed dataset were combined using Rubin’s Rule. Statistical analyses were performed with STATA version 16. For all analyses, a two-tailed *p*-value of < 0.05 was considered statistically significant.

## Results

**Fig. 1:**
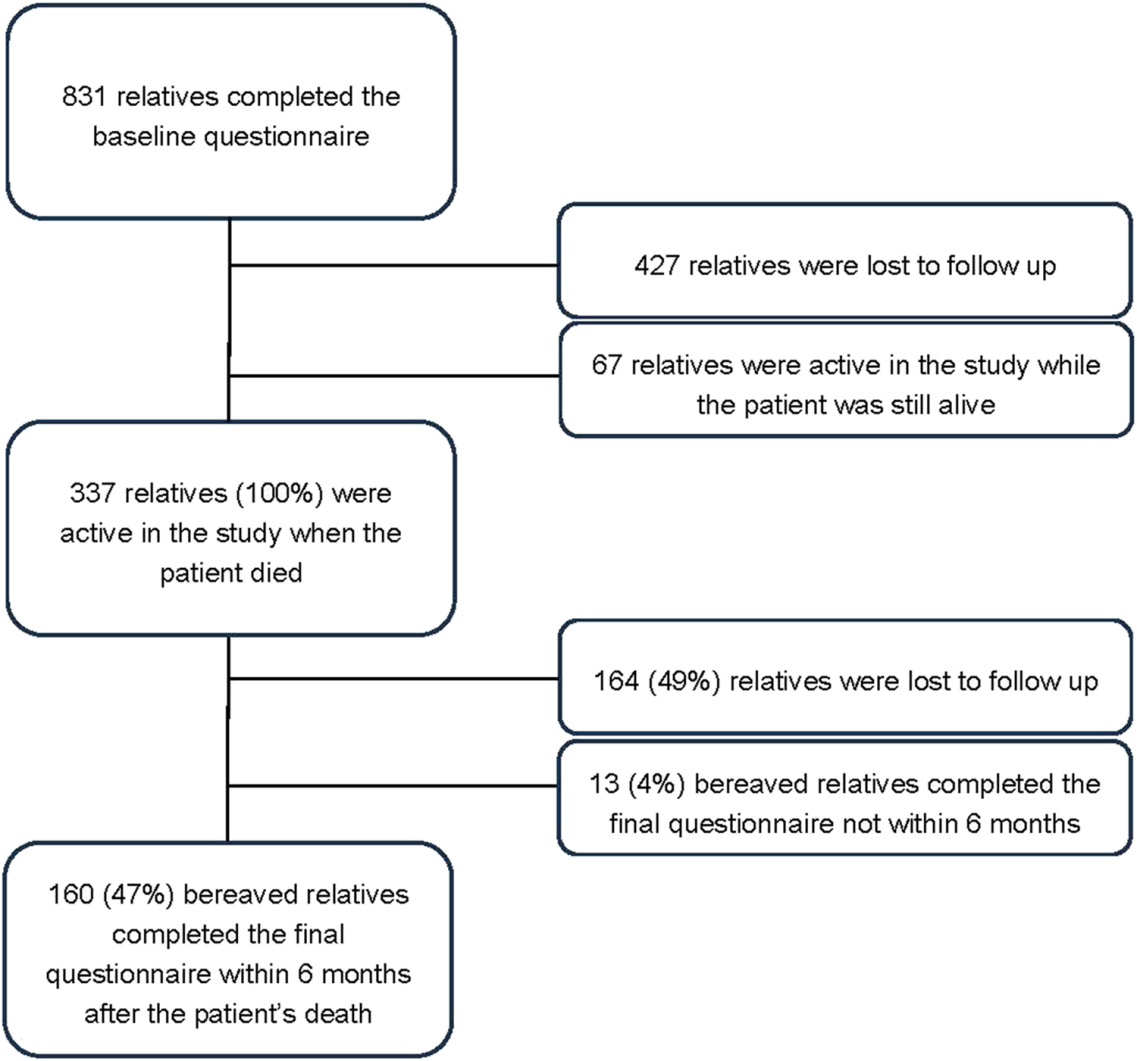
Flowchart inclusion

A total of 831 relatives completed the baseline questionnaire in the eQuiPe study. During the follow-up of the study, 337 relatives were still completing questionnaires at the time of the patient’s death. Of these, 173 bereaved relatives completed the final post-bereavement questionnaire; 160 did so within 6 months of the patient’s death (response rate = 47%). These 160 relatives were included in the analysis (Fig. 1).

### Bereaved relatives’ characteristics

The median age of the bereaved relatives was 65 years (range 25–85). Of the bereaved relatives, 56% were female and 35% had a high level of education. Most relatives (81%) had lost their partner and 14% had lost their parent. Half of the bereaved relatives had a high emotional functioning before bereavement (Table 1). The participating bereaved relatives did not significantly differ from other relatives in the eQuiPe study regarding their sociodemographic characteristics and baseline well-being (Supplementary Material 1).

**Table 1:** Characteristics of bereaved relatives (*n*=160)

		*N* (%)
Age ^a^	Median, range	65 (25-85)
Age﻿﻿ at time of patient’s death	18-54	36 (23%)
	55-63	35 (22%)
	64-69	37 (23%)
	≥70	51 (32%)
Gender	Male	70 (44%)
	Female	90 (56%)
Level of education	Low	30 (19%)
	Medium	74 (46%)
	High	56 (35%)
(Religious) worldview	No	63 (39%)
	Yes	97 (61%)
Type of relationship with patient ^b^	Partner	128 (81%)
	Child	22 (14%)
	Other*	9 (6%)
Level of EF	Median, range	67 (0-100)
EF before the patient’s death (0-100) ^c^	Low	72 (50%)
	High	71 (50%)

Variables may deviate from 100% due to rounding

Missing values: a: 1, b: 1 and c: 17

Abbreviations: *EF* Emotional functioning

### Openness of communication about illness and death

Overall, the average degree of open communication about illness and death between patients with advanced cancer and their relatives was 3.86 on a scale of 1 to 5 (*SE*=0.08, median=4, range: 1.8–5). Bereaved relatives most often reported that they did not avoid talking to the patient about their feelings and fears (71%) and that they did not find it difficult to talk about the patient’s illness because it might make the patient sad (69%). On the other hand, a quarter of the bereaved relatives reported that they were afraid to talk to the patient about continuing their life without the patient (25%) and 18% of the bereaved relatives reported that they rarely talked to the patient about the illness because they did not want to upset the patient (Table 2).

**Table 2:** Experienced open communication about illness and death as reported by bereaved relatives, based on the items of the CCID scale (*n*=158)

	(strongly) agree	neither agree nor disagree	(strongly) disagree
	*N* (%)	*N* (%)	*N* (%)
I was afraid to talk with the patient about continuing my life without him/her.	40 (25%)	19 (12%)	99 (63%)
I hardly talked with the patient about his illness because I did not want to make him/her sad.	28 (18%)	21 (13%)	109 (69%)
I avoided talking with the patient about his/her close death.	25 (16%)	26 (16%)	107 (68%)
I avoided talking with the patient about his/her feelings and fears.	20 (13%)	26 (16%)	112 (71%)
I didn’t know what to do or say to the patient in his/her suffering.	18 (11%)	35 (22%)	105 (66%)

Variables may deviate from 100% due to rounding

### Association between relatives’ characteristics and openness of communication

The degree of open communication about illness and death as perceived by the bereaved relatives was not associated with their age (*p*=0.745), gender (*p*=0.196), level of education (*p*=0.773; *p*=0.948), (religious) worldview (*p*=0.435), type of relationship with patient (child: *p*=0.837; other than partner: *p=*0.548), or level of emotional functioning before the patient’s death (*p*=0.075) (Table 3).

**Table 3:** Association between relative characteristics and degree of open communication in bereaved relatives of patients with advanced cancer in univariate and multivariable regression

Variables	Univariate analysis *(n*=160)			Multivariable analysis (*n*=160)		
	β	[95% CI]	*P*-value	β	[95% CI]	*P*-value
Age						
Age at time of patient’s death	.002	-.011 – .015	0.75	.002	-.017 – .020	0.85
Gender						
Male [reference]						
Female	-.203	-.511 – .106	0.20	-.187	-.524 – .150	0.28
Level of education						
Low [reference]						
Medium	.062	-.359 – .482	0.77	.120	-.306 – .547	0.58
High	.015	-.426 – .455	0.95	.090	-.363 – .543	0.70
(Religious) worldview						
No [reference]						
Yes	-.125	-.439 – .190	0.44	-.114	-.456 – .228	0.51
Type of relationship with patient						
Partner [reference]						
Child	.047	-.400 – .494	0.84	.097	-.542 – .735	0.77
Other	.214	-.489 - .917	0.55	.189	-.569 – .947	0.62
Level of EF						
EF before the patient’s death	.006	-.001 – .012	0.08	.005	-.002 – .013	0.17

Missings: Missing values were imputed: age (*n*=1), type of relationship with the patient (*n*=1), the degree of open communication (*n*=2), and emotional functioning before the patient’s death (*n*=17)

Abbreviations: *EF* Emotional functioning. *CI* Confidence interval

*p < 0.05

### Association between openness of communication and bereavement distress

The degree of open communication as perceived by the bereaved relatives was negatively associated with bereavement distress (β=-2.990, *p*=0.003), indicating that a higher degree of open communication was associated with a lower degree of bereavement distress, adjusted for age, gender, level of education, (religious) worldview, type of relationship with the patient and emotional functioning before the patient’s death (Table 4).

**Table 4:** Association between openness of communication and bereavement distress as perceived by bereaved relatives of patients with advanced cancer

	β	95% CI	*P*-value
Degree of open communication	-2.990	-4.977 – -1.003	0.00*
*Confounders*			
Age			
At time of patient’s death	.075	-.156 – .305	0.52
Gender			
Male [reference]			
Female	4.841	.667 – 9.014	0.02*
Level of education			
Low [reference]			
Medium	-1.502	-6.755 – 3.751	0.57
High	-4.663	-10.213 – .887	0.10
(Religious) worldview			
No [reference]	2.784	-1.401 – 6.968	0.19
Yes			
Type of relationship with patient			
Partner [reference]			
Child	-8.814	-16.703 – -.926	0.03*
Other	-9.462	-18.236 – -.688	0.04*
Level of EF			
EF before the patient’s death	-.172	-.264 – -.079	<0.01*

Missings: Missing values for completed IES-R were replaced by the group mean for the item. Other missing values were imputed: age (*n*=1), type of relationship with the patient (*n*=1), degree of open communication (*n*=2), emotional functioning before the patient’s death (*n*=17), and degree of bereavement distress (*n*=1)

Abbreviations: *EF* Emotional functioning. *CI* Confidence interval

*p < 0.05

## Discussion

The majority of bereaved relatives of patients with advanced cancer reported a high degree of open communication about illness and death with the patient during the last three months of the patient’s life. Relatives’ age, gender, level of education, (religious) worldview, type of relationship with the patient, and level of emotional functioning before the patient’s death were not associated with the degree of open communication. However, a higher degree of open communication was associated with a lower degree of bereavement distress in the bereaved relatives.

A high degree of open communication (3.86 on a scale of 1 to 5) between relatives and patients was found and this suggests that many relatives have little or no difficulty communicating about illness and death with the patient and do not avoid these conversations. Dutch relatives show a relatively higher degree of openness of communication compared to other studies, which showed a low to moderate degree of open communication among Chinese relatives [20], Israeli relatives [7, 24] and also among Danish relatives [11]. It is important to note that our study population was relatively homogeneous in terms of cultural background. Therefore, the results cannot be generalized to all patients with advanced cancer in the Netherlands, as it is known that within certain cultures, e.g. the Muslim culture in the Netherlands, it is also not common to talk openly about death and dying [17]

Surprisingly, we found no association between the degree of open communication and any of the relatives’ characteristics, suggesting that the degree of open communication is independent of the relative’s age, gender, level of education, (religious) worldview, type of relationship with the patient, and level of emotional functioning. We expected that female relatives report a higher degree of open communication compared to male relatives [7, 22, 23], although other studies have also been inconclusive regarding gender [7, 24]. The differences in results observed between studies may be due to differences in the study populations examined, such as the inclusion of relatives with different relationships to the patient (beyond spouses) or different age groups, as well as potential differences in the research methods used.

A higher degree of open communication during the last three months of the patient’s life was associated with a lower degree of bereavement distress, adjusted for relatives’ age, gender, level of education, (religious) worldview, type of relationship with the patient, and level of emotional functioning before the patient’s death. This finding supports previous research indicating that open communication about the end of life is associated with less negative bereavement outcomes among relatives [11–13].

Although open communication has been associated with less negative bereavement outcomes, it is important to keep in mind that communication is complex and not all patients and relatives are willing or are able to communicate openly about illness and death. According to a systematic review conducted by Hasson-Ohayon et al. [34], the benefits of open communication depend on factors such as the responsiveness of the other person, the synchronicity of communication needs, contextual factors, and personal status. For example, Parker et al. [35] found in their systematic review that the information needs of patients and relatives diverged over time, with relatives wanting more information and patients wanting less. These conflicting needs may also contribute to the distress experienced by patients and their relatives as they cope with illness and death. In addition, the degree of bereavement distress is also known to be complex, with poor physical health, lower perceived social support, family difficulties in accepting the death, and the location of the death being other known risk factors [36]. The result is a complex and multifactorial association that needs to be further explored.

## Study limitations

Some methodological limitations of our study need to be addressed. First, response bias may arise, as those who are more open in their in communication may be more inclined to participate in the post-bereavement questionnaire after the patient's death, while those who were highly distressed or had less open communication might opt out of completing the final questionnaire. This could result in an overestimation of the degree of open communication about illness and death. Regarding response bias, it is important to mention the high number of relatives who were lost to follow-up (49%). The reasons for this are unknown, but it might be possible that the burden on relatives and the sensitive nature of the topic of end-of-life communication has played a role. Secondly, there may be a selection bias because the relatives were chosen by the patients themselves. Third, we were unable to assess the differences in cultural and religious factors in our study due to the majority of bereaved relatives sharing the same background. Therefore, caution should be exercised when generalizing the results of our study to the broader Dutch population. Last, relatives were asked to complete a short final questionnaire within six months after the patient’s death. Their perspectives might have changed in this period, and recall bias cannot be ruled out.

## Clinical implications

Our findings suggest that open communication about illness and death between patients and their relatives plays an important role at the end of a patient's life. It is important for palliative care professionals to recognize the potential benefits of open communication between patients and relatives, although further understanding of this causal relationship is needed. The first step for professionals could be encouraging patients and their relatives to express their thoughts, concerns and preferences in order to facilitate meaningful dialogue. As open communication will not be welcomed by all patients and relatives (due to religion, culture or other factors), an individualized approach is needed.

In addition, public awareness about illness and death may result into a higher degree of open communication about illness and death between patients and their relatives. At this moment, the Dutch government prioritizes education and awareness campaigns to bring death and dying back into society. One example is the national SIRE campaign, which was created to inspire people to talk about death by showing disarming conversations between influencers and their loved ones. In doing so, we pave the way for a more supportive society in which open communication becomes an integral part of the process for patients and their relatives facing illness and death.

## Conclusion

The majority of relatives of patients with advanced cancer experience open discussions about illness and death with the patient during the last three months of the patient’s life. A higher degree of open communication is associated with less bereavement distress for the relatives. This information can be used as input for further research to explore the benefits of open communication between patients and relatives at the end of life.

### Supplementary information

Supplementary file 1 Characteristics of bereaved relatives (DOCX 15 kb)

## Data Availability

Since 2011, PROFILES registry data is freely available according to the FAIR (Findable, Accessible, Interoperable, Reusable) data principles for non-commercial (international) scientific research, subject only to privacy and confidentiality restrictions. The datasets analyzed during the current study are available through Questacy (DDI 3.x XML) and can be accessed by our website (www.profilesregistry.nl). In order to arrange optimal long-term data warehousing and dissemination, we follow the quality guidelines that are formulated in the “Data Seal of Approval” (www.datasealofapproval.org) document, developed by Data Archiving and Networked Services (DANS). The data reported in this manuscript will be made available when the eQuiPe study is completed.
