# Supplementary material for: Open communication between patients and relatives about illness & death in advanced cancer—results of the eQuiPe Study
Source: Support Care Cancer. 2024 Mar 6;32(4):214. doi: 10.1007/s00520-024-08379-5 (PMC10917842; doi:10.1007/s00520-024-08379-5)
Supplement: Supplementary file 1 — Characteristics of bereaved relatives (DOCX 15 kb) [file 520_2024_8379_MOESM1_ESM.docx]

**Supplementary material 1**

*Characteristics of bereaved relatives*

|  |  | Characteristics of included bereaved relatives | Characteristics of all relatives at baseline |
| --- | --- | --- | --- |
|  |  | *N=160* | *N= 831 (831-160)* |
|  |  | *N* (%) | N (%) |
| Age ^a^  At time of  patient’s death | Median, range  18-54  55-63  64-69  ≥70 | 65 (25-85)  36 (23%)  35 (22%)  37 (23%)  51 (32%) | 63 (18-87)  227 (27%)  182 (22%)  188 (23%)  234 (28%) |
| Gender | Male  Female | 70 (44%)  90 (56%) | 324 (39%)  507 (61%) |
| Level of education | Low  Medium  High | 30 (19%)  74 (46%)  56 (35%) | 208 (25%)  373 (45%)  243 (29%) |
| (Religious) worldview | No  Yes | 63 (39%)  97 (61%) | 327 (40%)  497 (60%) |
| Type of relationship with patient ^b^ | Partner  Child  Other* | 128 (81%)  22 (14%)  9 (6%) | 612 (74%)  140 (17%)  79 (9%) |
| Level of EF  Before the   patient’s death   (0-100) ^c^ | Median, range  Low  High | 67 (0-100)  72 (50%)  71 (50%) | 75 (0-100)  373 (47%)  418 (53%) |

*Abbreviations: EF: Emotional functioning.
Variables may deviate from 100% due to rounding.
Missing values: a: 1, b: 1 and c: 17.*
